# The emerging role of epigenetic regulation in the progression of silicosis

**DOI:** 10.1186/s13148-022-01391-8

**Published:** 2022-12-09

**Authors:** Haoyu Yin, Yujia Xie, Pei Gu, Wei Li, Yingdie Zhang, Yuxin Yao, Weihong Chen, Jixuan Ma

**Affiliations:** 1 Department of Occupational and Environmental Health, School of Public Health, Tongji Medical College, Huazhong University of Science and Technology, Wuhan, 430030 Hubei China; 2 Key Laboratory of Environment and Health, Ministry of Education & Ministry of Environmental Protection, and State Key Laboratory of Environmental Health (Incubating), School of Public Health, Tongji Medical College, Huazhong University of Science and Technology, Wuhan, 430030 Hubei China; 3 Key Lab of Environment and Health, School of Public Health, Xuzhou Medical University, Xuzhou, 221004 Jiangsu China

**Keywords:** Silicosis, Epigenetic, DNA methylation, ncRNA, Histone modification

## Abstract

Silicosis is one of the most severe occupational diseases worldwide and is characterized by silicon nodules and diffuse pulmonary fibrosis. However, specific treatments for silicosis are still lacking at present. Therefore, elucidating the pathogenesis of silicosis plays a significant guiding role for its treatment and prevention. The occurrence and development of silicosis are accompanied by many regulatory mechanisms, including epigenetic regulation. The main epigenetic regulatory mechanisms of silicosis include DNA methylation, non-coding RNA (ncRNA), and histone modifications. In recent years, the expression and regulation of genes related to silicosis have been explored at epigenetic level to reveal its pathogenesis further, and the identification of aberrant epigenetic markers provides new biomarkers for prediction and diagnosis of silicosis. Here, we summarize the studies on the role of epigenetic changes in the pathogenesis of silicosis to give some clues for finding specific therapeutic targets for silicosis.

## Background

Silicosis is a potentially pulmonary interstitial disease caused by long-term exposure to crystalline silica dust (aerodynamic diameter < 10 μm) [1]. Due to its high morbidity and mortality, silicosis continues to be a public health issue worldwide [2], especially in developing countries such as China, India, Vietnam, and Brazil [3]. According to a report from the Global Burden of Disease (GBD) research in 2017, the number of silicosis incidents increased from 14, 973 in 1990 to 23, 695 in 2017 [4]. In particular, the highest number of incident cases and the highest measure of age-standardized incidence rate (ASIR) were counted in China (Taiwan), followed by Papua New Guinea, and then China (mainland) [4]. Recent outbreaks of silicosis in the mining industry in the USA and Australia demonstrated that even in developed countries, it is necessary to be vigilant in the control of dust levels [5].

Occupational exposure to respirable crystalline silica usually happens when a substance or material containing silica is mechanically disintegrated [6]. Long-term inhalation of respirable crystalline silica can cause silicosis, which is characterized by persistent inflammatory response, diffuse interstitial pulmonary fibrosis, and the formation of silicon nodules, ultimately leading to impaired lung function, respiratory failure, or even death [5]. Therefore, silicosis is a progressive disease, and early prevention is of great significance. Unfortunately, despite extensive research into the mechanisms of silicosis, there are still no effective drugs or treatments to reverse or halt the progression of silicosis to date.

Epigenetics is currently one of the fastest growing fields in biological research. It refers to the heritable changes of gene expression without altering the DNA sequence, which eventually leads to the changes of function and phenotype [7]. Epigenetic processes mainly include methylation modification, non-coding RNA (ncRNA), histone modifications, genomic imprinting, and chromatin remodeling [8]. These processes modify gene expression and, due to their chemical modifications, affect the transmission of gene activity from one generation of cells to the next, providing an alternative mechanism for biological inheritance and variation [9]. Previous studies have shown that epigenetics plays a crucial role in many cellular processes, such as the regulation of gene expression and transcription, cell growth and differentiation, and chromosome remodeling and inactivation [10]. Recent evidence showed that epigenetic mechanisms could act as a link between environmental stimuli and gene expression, suggesting that epigenetic modifications are the adaptation of genes in response to environmental changes [11]. Epigenetic regulations, especially methylation modification, ncRNA regulation, and histone modification, have been reported to be associated with the progression of silicosis [12]. In this review, we will comprehensively summarize the pathogenesis of silicosis from the perspective of epigenetic modifications, with the aim of providing new ideas for early diagnosis, disease assessment, and targeted treatment of silicosis.

## Methylation modification

### DNA methylation

DNA methylation is one of the most intensively studied epigenetic modifications. DNA methylation is catalyzed by a family of DNA methyltransferases (DNMTs), which transfer a methyl group from S-adenyl methionine (SAM) to the fifth carbon of cytosine residue of CpG dinucleotide to form 5-methylcytosine [12]. A great number of studies have demonstrated that DNA methylation can cause changes in chromatin structure, DNA conformation and stability, and the way of interaction between DNA and protein, thus controlling gene expression [13].

Most changes in methylation patterns exist in the process of cell division and differentiation, in which new DNA methylation, demethylation, and maintenance of methylation marks may occur [14]. DNA methylation mainly exists in gene bodies, intergenic regions, DNA repetitive sequences, and endogenous retrotransposons. In contrast, CpG-rich regions in the genome, called CpG islands, are unaffected by DNA methylation; they are consistent with promoters, replication origins, and cis-regulatory transcription elements [15]. DNA methylation is catalyzed by three DMNTs: DNMT1, DNMT3a, and DNMT3b. DNMT1 regulates the maintenance of DNA methylation during cell division and preferentially selects hemimethylated DNA as its substrate, while DNMT3a and DNMT3b regulate de novo DNA methylation and establish new DNA methylation modes [16]. These enzymes have various regulatory functions and participate in many biological processes such as cell proliferation, organ development, aging and tumorigenesis [17].

Studies have shown that DNA methylation changes at the genome-wide level are related to silica-induced pulmonary fibrosis.

#### In vitro and in vivo experiments

In an alveolar macrophage/fibroblast co-culture system, Li et al. conducted the genome-wide DNA methylation profiles and observed that fibroblasts presented extensive methylation changes when macrophages were exposed to silica. Those methylation changes were mainly involved in the mitogen-activated protein kinase (MAPK) signaling pathway and metabolic pathways [18]. In the study by Wang et al., they found that transforming growth factor-β1 (TGF-β1) induced global DNA methylation in fibroblasts in a transforming growth factor-β receptor I (TGFBR1)/Smad3-dependent manner. In addition, methyl-CpG-binding domain 2 (Mbd2) selectively bound to methylated CpG DNA within the erythroid differentiation regulator 1 (Erdr1) promoter to inhibit its expression, which promoted fibroblasts to differentiate into myofibroblasts by enhancing TGF-β/Smads signaling transduction and exacerbating silica-induced pulmonary fibrosis [19].

Confirmed by in vivo experiments, Li et al. ascertained a dose-dependent decrease in genomic methylation of fibroblasts during silica-induced transdifferentiation. These genes mainly focused on metabolism, environmental information processes, cellular processes, and biological systems [18]. In another research, Zhang et al. reported that DNMTs increased in the tissues of silica-exposed rats, and pretreatment with DNMT inhibitor 5-aza-dC could reduce the expression of collagen type I (COL-I), collagen type II (COL-III), and alleviate silica-induced pulmonary fibrosis [20].

#### Human experiment

The abnormal levels of genome-wide DNA methylation were also observed in subjects with silicosis. Zhang et al. reported that the methylation level of CpG loci in lung tissues of silicosis was approximately 17% higher than that of healthy controls [21].

In addition to genome-wide DNA methylation, the dysregulation of specific genes-methylation in silicosis patients was also observed in previous studies. Studies found that DNA methylation could be directly involved in the regulation of gene expression during the pathological process of silicosis. DNA methylation status in the promoter regions of five tumor suppressor genes (e.g., *MGMT*, *p16INK4a*, *RASSF1A*, *DAPK*, *RARb*) was found to be higher in the serum of silicosis patients than that in healthy controls [22]. By analyzing the DNA methylation spectrum in the lung tissue of silicosis, Zhang et al. revealed that the number of phosphatase and tensin homolog deleted on chromosome ten (PTEN) and c-Jun CpG promoter methylated sites were increased in the advanced stage of silicosis, and the hypermethylation of PTEN promoter was associated with the decreased expression of PTEN protein [21].

DNA methylation does exist in the process of silicosis, but according to the current research, a large number of in vivo and in vitro experiments and epidemiological studies are still needed to comprehensively evaluate its specific changes and effects. There are few studies on the role of DNA methylation in silicosis, but the research on silica-induced pulmonary fibrosis can be used as a reference.

### RNA methylation

Until now, more than 150 post-transcriptional modifications have been discovered in RNA in all organisms [23]. N^6^-methyladenosine (m^6^A) is the most common and abundant RNA modification in eukaryotic cells [24]. m^6^A RNA methylation mainly occurs in the common motif of RRm^6^ACH (R = A/G and H = A/C/U), which is enriched in the 3′ untranslated regions (3′UTRs), around the stop codons, and within the internal long exons [25, 26]. m^6^A RNA methylation modification has been proved to play significant roles in regulating RNA metabolism at the post-transcriptional level, including the processing, transmission and translation of mRNA, and the biogenesis of lncRNA and miRNA [27, 28]. Besides, m^6^A RNA methylation has shown a variety of key roles in mammals, such as the renewal of stem cell, embryonic development, immunity, sex determination, and tumorigenesis [29]. Many studies on m^6^A RNA methylation have demonstrated that the regulatory mechanisms of m^6^A RNA methylation are involved in a lot of human diseases, including heart failure, diabetes, viral hepatitis, especially in human cancers [30–32].

Luo et al. indicated that m^6^A RNA methylation was abnormal in the early inflammatory stage of silicosis, and m^6^A methylation modification of cireSLC2A13 was involved in the activation of macrophages in the above process [33]. Using an m^6^A-circRNA epitranscriptomic chip, Wang et al. found that the screened hsa_circ_0000672 and hsa_circ_0005654 were explicitly participated in silica-induced pulmonary fibrosis by targeting eukaryotic translation initiation factor 4A3 (EIF4A3), suggesting that m^6^A RNA methylation of circRNAs mediated silica-induced fibrosis [34]. Besides, Sun et al. demonstrated that alkB homologue 5 (ALKBH5), a well-known m^6^A demethylase, was increased in the lung tissues of silica-inhaled mice. And ALKBH5 promoted silica-induced lung fibrosis through miR-320a-3p/forkhead box protein M1 (FOXM1) pathway or targeting FOXM1 directly. This finding indicated that methods targeting ALKBH5 might be effective in the treatment of pulmonary fibrosis [35]. Similarly, the m^6^A methylation regulator methyltransferase-like 3 (METTL3) can be considered as an important biomarker for diagnosing pulmonary fibrosis occurrence because of its low expression in pulmonary fibrosis [36].

Although there are few reports on the role of RNA methylation in silicosis, the existing researches have proved that RNA methylation plays a vital role in the occurrence and development of silicosis. With the advancement of detection, the biological functions, potential molecular mechanisms, regulatory factors, and downstream target genes of RNA methylation will be further understood.

## ncRNA

non-coding RNA (ncRNA) is a kind of abundant RNA that does not encode proteins. According to the length, shape and location, ncRNAs are divided into different types, mainly including microRNAs (miRNAs), long non-coding RNAs (lncRNAs), circular RNAs (circRNAs), small interfering RNAs (siRNAs), piwi-interacting RNAs (piRNAs), and small nuclear RNAs (snRNAs) [37]. ncRNAs can serve as the functional regulatory factors to regulate cellular processes, including chromatin remodeling, gene transcriptional regulation, and signal pathway activation or inhibition. The network of ncRNAs can influence multiple molecular targets to drive specific cellular biological reactions. Therefore, ncRNAs act as key regulators in the occurrence and development of diseases, dysregulation of ncRNA has been reported to be linked with a diversity of diseases, such as neurological, cardiovascular, respiratory disorders, and cancer [38].

### miRNA

miRNAs are highly conserved non-coding single-stranded RNA molecules with the lengths of 18–22 nucleotides. They mainly mediate post-transcriptional gene silencing by destroying the stability or inhibiting the translation of target mRNA [39]. It is estimated that miRNAs can regulate the translation of more than 60% of protein-coding genes [40]. miRNAs have multiple functions, including the regulation of cell proliferation, differentiation and apoptosis, and tissue development [41, 42].

#### In vitro and in vivo experiments

With the development of silica-induced pulmonary fibrosis, Han et al. found that the expression of miR-449a was decreased, which could negatively regulate the target gene B-cell lymphoma-2 (Bcl-2), trigger autophagy and inhibit the proliferation of fibroblasts, therefore playing a role in inhibiting pulmonary fibrosis [43]. Study conducted by Yuan et al. clarified that miR-770-5p played an anti-fibrotic effect in silica-induced pulmonary fibrosis by targeting TGFBR1 [44]. And overexpressed miR-490-3p could prevent the process of fibroblast-to-myofibroblast transition (FMT) in vitro [45]. Furthermore, Qian et al. uncovered that miR‑29c expression was significantly downregulated in the lungs of silicotic rats and in the pulmonary fibroblasts of the in vitro model of silicosis, while miR‑29c overexpression significantly suppressed the expression levels of fibrosis‑related genes, such as α‑smooth muscle actin (α‑SMA), COL-I, and COL-III [46]. Chen et al. observed that miR-155-5p negatively regulated meprin α, a major regulator of anti-fibrotic peptide N-acetyl-seryl-aspartyl-lysyl-proline. And treatment with anti-miR-155-5p could elevate meprin α, ameliorate the activation of macrophage and fibroblast, and attenuate lung fibrosis in mice induced by silica. Sustained suppression of meprin α and beneficial effect of its rescue by inhibition of miR-155-5p indicated that miR-155-5p and meprin α were two primary regulators of silicosis [47]. In addition, the group of Niu et al. confirmed that the increase of miR-7219-3p facilitated the FMT process, as well as cell proliferation and migration, while the inhibition of exosomal miR-7219-3p partially suppressed FMT and silica-induced pulmonary fibrosis [48].

It has been well recognized that the endothelial mesenchymal transition (EMT) plays a crucial role in the process of silicosis [49, 50]. More importantly, the effects of miRNA-regulated EMT in silicosis have been widely identified in previous studies. Xu et al. observed that miR-29b-2-5p and miR-34c-3p were significantly reduced in lung epithelial cells treated with TGF-β1 and mouse silicosis models. And overexpression of miR-29b-2-5p or miR-34c-3p inhibited the EMT process and abrogated the profibrotic effect in vitro [51], indicating that the upregulation of miR-29b-2-5p and miR-34c-3p could play a protective role in silicosis. In addition, Yu et al. found that let-7d negatively regulated silica-induced EMT and inhibited silica-induced pulmonary fibrosis, which might be partially achieved through direct binding to high mobility group protein 2 (HMGA2) [52].

The miRNA expression profiles of experimental silicosis rats identified 14 upregulated and 25 downregulated miRNAs in lung samples, of which miR-299 increased the most (7.28-fold changes) and miR-375 decreased the most (0.23-fold changes) [53]. Similarly, studies found that the up-regulation of miRNA-423-5p and miR‑146a and the down-regulation of miR-503, miRNA-26a-5p and miR‑181b in silicosis rats might be related to the occurrence and development of early silicosis [54–56].

In the study of Gao et al., decreased level of miR-411-3p was found in silicosis rats. And the increased miR-411-3p expression could abrogate silicosis by decreasing the ubiquitination degradation of Smad7 by Smad ubiquitination regulator 2 (Smurf2) and blocking the activation of TGF-β/Smad signaling [57]. To explore the potential function of miR-542-5p in silicosis, Yuan et al. established a silicosis mouse model by intratracheal instillation of silica suspension and found that miR-542-5p was significantly reduced in the fibrotic lung tissues. They further proved that the upregulation of miR-542-5p visually attenuated a series of fibrotic lesions, including alveolar structural damage, alveolar interstitial thickening, and silica-induced nodule formation, suggesting that miR-542-5p might be a new therapeutic target for silicosis [58]. Similarly, the increased expression of miR-326 attenuated pulmonary fibrosis in mice exposed to silica, and the relevant mechanism might be that miR-326 inhibited inflammation and promoted autophagy activity through the target protein tumor necrosis factor superfamily 14 (TNFSF14) and polypyrimidine tract-binding protein 1 (PTBP1), respectively [59]. Furthermore, by injecting miR-489 agomir via the tail vein into mice, Jin et al. confirmed that the upregulation of miR-489 had a potential therapeutic effect in silica-induced pulmonary fibrosis, which was related to the inhibition of TGF-β1 release [60]. In addition, the expression of miR-1224-5p was significantly upregulated in lung tissue with silica-induced pulmonary fibrosis. miR-1224-5p could directly inhibit the expression of the target gene beclin 1 (BECN1), which in turn blocked the translocation of Parkinson’s disease protein 2 (PARK2) to mitochondria, resulting in mitochondrial damage and promoting pulmonary fibrosis [61].

By establishing the silicosis model in mice, Qi et al. reported that miR-34a expression was downregulated in the fibrotic lung tissues induced by silica exposure. Overexpression of miR-34a markedly inhibited EMT, which might be achieved in part by targeting Smad4 [62]. Furthermore, using microarray assay, Sun et al. discovered that miRNA-29b was dynamically downregulated by silica and effected the promotion of EMT. Delivering miRNA-29b to mice could significantly inhibit silica-induced EMT, prevent lung fibrosis, and improve lung function [63]. Ji et al. observed that the expression of miR-486-5p was markedly downregulated in both silicosis patients and silicosis mice models. Similar to miR-31, miR-200 and miR-29 [64–66], overexpression of miR-486-5p could alleviate silica-induced pulmonary fibrosis in mice [67].

#### Human experiment

Yang et al. collected total RNAs from the peripheral blood leukocytes of 23 silicosis patients and 23 healthy controls, and the different miRNAs were screened using microarrays, the results showed that miR-19a in peripheral blood leukocytes could be used as an effective biomarker for silicosis [68]. Rong et al. found that miR-200c and miR-29c were decreased in subjects with severe lung function decline, and the abnormal extracellular matrix regulated by these miRNAs might play a crucial role in the decline of lung function among subjects with silicosis [69] (Table 1).

**Table 1:** The main miRNA changes involved in silicosis

miRNA	Tissue/Cell/Patients	Expression level	Target gene	References
miR-449a	Mice NIH-3T3	Down	Bcl-2	[43]
miR-770-5p	MRC-5	Down	TGFBR1	[44]
miR-490-3p	MRC-5, NIH/3T3	Down	TGFBR1	[45]
miR‑29c	Rats Pulmonary fibroblasts Rat NR8383 Pulmonary macrophages	Down	TGF‑β1/α‑SMA	[46]
miR-155-5p	MEFs	Up	Mep1a	[47]
miR-7219-3p	Mice RAW264.7, HEK-293T, NIH-3T3	Up	Spouty1	[48]
miR-29b-2-5p	Mice A549, BEAS-2B	Up	MEKK2/NOTCH2	[51]
miR-34c-3p	Mice A549, BEAS-2B	Up	MEKK2/NOTCH2	[51]
let-7d	A549, THP-1	Up	HMGA2	[52]
miR-299	Rats	Up		[53]
miR-375	Rats	Down		[53]
miRNA-423-5p	Rats	Up		[54]
miRNA-26a-5p	Rats	Down		[54]
miR-146a	Rats	Up	Interleukin-1β	[55]
miR‑181b	Rats	Down	TNF-α	[55]
miR-503	Mice HBE, A549	Down	PI3K	[56]
miR-411-3p	Rats fibroblasts isolated from the lungs of newborn rats	Down	TGF-β/Smad	[57]
miR-542-5p	Mice	Down	ITGA6	[58]
miR-326	Mice HBE, A549, MRC-5, NIH/3T3	Up	TNFSF14/PTBP1	[59]
miR-489	Mice	Up	TGF-β1	[60]
miR-1224-5p	Mice NIH-3T3, MRC-5	Up	BECN1	[61]
miR-34a	Mice	Up	Smad4	[62]
miR-29b	Mice RLE-6TN	Up	COL-I/COL-III	[63]
miR-31	Mice MRC-5	Down	integrin α(5)/RhoA	[64]
miR-200	Rats MRC-5, RLE-6TN	Down	TGF-β1	[65]
miR-29	Mice	Down	TGF-β	[66]
miR-486-5p	Mice Patients	Down	Smad2/COL6A6	[67]
miR-19a	Patients	Down		[68]
miR-200c	Patients	Down		[69]
miR-29c	Patients	Down		[69]

### lncRNA

lncRNAs are more than 200 nucleotides in length, which have almost no protein coding ability. lncRNAs can be classified as antisense lncRNAs, intronic lncRNAs, lincRNAs, promoter-associated lncRNAs, UTR-associated lncRNAs et al. [70].

Generally, lncRNAs regulate gene expression mainly via the following 5 ways: (1) at the epigenetic level, lncRNAs regulate the epigenetics of organisms through DNA methylation, histone modification, chromosome silencing, and genomic imprinting [71]; (2) at the transcriptional level, lncRNAs can bind to transcription factors through cis or trans regulation to change RNA activity and control gene transcription, as well as complementary pairing with DNA and RNA bases to mask splice sites, promoters, and miRNA binding sites, thereby altering gene expression or protein function [72]; (3) lncRNAs can directly participate in the splicing of mRNA precursors; (4) lncRNAs can bind miRNAs as competitive endogenous RNAs (ceRNAs) and participate in the regulation of target genes [73]; (5) lncRNAs bind to different proteins through spatial structure, allowing direct protein action and enhancing protein stability [74].

#### In vitro and in vivo experiments

To identify the crucial lncRNA-mRNA networks for silica-induced pulmonary fibrosis, Lei et al. selected a total of 1140 differently expressed mRNAs (DEmRNAs) and 1406 differently expressed lncRNAs (DElncRNAs), in which they demonstrated that lncRNA AK131029 was specifically overexpressed in silicosis [75]. Using high throughput mRNA sequencing, Chen et al. reported that miR-455-3p and five lncRNAs (LOC105375913, NEAT1, LOC105375181, LOC100506098, and LOC105369370) related ceRNA network might be the toxicity mechanism of microcrystalline silica particles to human airway epithelial cells (AEC), which might provide a method for the studies of the pathogenesis of early silicosis [76].

One study found that lncRNA cardiac hypertrophy-related factor (CHRF) was upregulated in silica-induced pulmonary fibrosis, along with RAW264.7 cells and NIH-3T3 cells treated with silica particles. CHRF could be used by adsorption of miR-489 on the target genes myeloid differentiation factor 88 (MyD88) and Smad3, which activated inflammatory and fibrotic signaling pathways and promoted the development of pulmonary fibrosis induced by silica particles [77]. Li et al. revealed that the upregulated lncRNA XIST could regulate miR-101-3p, which in turn upregulated the expression of E-box-bound zinc finger protein 1 (ZEB1), the transcription factor in the EMT process, thereby promoting the EMT process in alveolar epithelial cells during silicosis-associated pulmonary fibrosis EMT [78]. Furthermore, Liu et al. demonstrated that silica-stimulated macrophages secreted TGF-β1 to induce lncRNA activated by transforming growth factor-β (lncRNA-ATB) in epithelia cells, promoting EMT by binding with miR-200c and releasing ZEB1 [79].

Using microarray assays, Sai et al. investigated the changes of lncRNAs expression in lung tissue of silica-exposed rats, and observed that silica exposure led to altered expression profiles of 682 lncRNAs (300 upregulated and 382 downregulated). Among them, 73 ceRNA pairs were identified through predictive analysis [80]. By evaluating the differential expression of lncRNAs in the lungs of control and silicosis rats using RNA-sequencing, Cai et al. found that a total of 306 lncRNAs were differentially expressed in the lungs of silicotic rat, including 224 upregulated and 82 downregulated lncRNAs, in which LOC103691771 played a major role in myofibroblast differentiation, and might be a potential therapeutic target for silicosis [81].

Combined with alleviating the fibrotic effects of miR-326 in silica particle-exposed mice model, Wu et al. found that lnc-SNHG1 remarkably adsorbed miR-326 and promoted specificity protein 1 (SP1) expression, thereby accelerating the conversion of fibroblasts to myofibroblasts and synergistically promoting the development of pulmonary fibrosis [82].

Wang et al. found that there were 1077 differentially expressed lncRNAs (378 upregulated and 699 downregulated) between normal and silicotic rats, and MRAK050699 knockdown inhibited EMT via regulating the TGF-β/Smad3 signaling pathway. This finding indicated that MRAK050699 played an important role in EMT and could be served as a potential therapeutic target for silicosis [83]. Furthermore, in a silica particle-induced lung fibrosis mouse model, lncRNA MALAT1, as a molecular “sponge”, could adsorb miR-503, reduce the expression of miR-503, and activate the PI3K/Akt/Snail signaling pathway, leading to EMT and triggering lung fibrosis [56]. lncRNA MALAT1 could also affect the expression of miR-145, which directly targets TGFBR2 and Smad3 to inhibit the EMT process. Consequently, lncRNA MALAT1 could induce the onset of EMT and promote the process of lung fibrosis by adsorbing miR-145 [84]. In addition, a previous study established a silicosis mouse model and an in vitro EMT model of A549 cells and found that lncRNA UCA1 might regulate the EMT process by competitively adsorbing miR-204-5p to release its target gene ZEB1 [85].

#### Human experiment

Ma et al. found that the expression of lncRNA-ATB was upregulated in the plasma of coal miners and was positively correlated with the concentration of TGF-β [86]. Besides, the RNA-sequencing data were comprehensively analyzed in the peripheral blood lymphocytes of eight participants (four silicosis cases *vs.* four healthy controls), the results showed that the expression of lncRNA ADGRG3 was low in silicosis patients, and the relevant mechanism might be that single nucleotide polymorphisms rs1814521 in lncRNA ADGRG3 was associated with the susceptibility of silicosis [87] (Table 2).

**Table 2:** The main lncRNA changes involved in silicosis

lncRNA	Tissue/Cell/Patients	Expression level	Target gene	References
lncRNA AK131029	Beas-2B	Up		[75]
LOC105375913	AEC	Up		[76]
NEAT1	AEC	Up		[76]
LOC105375181	AEC	Up		[76]
LOC100506098	AEC	Up		[76]
LOC105369370	AEC	Up		[76]
lncRNA CHRF	Mice RAW264.7, NIH-3T3	Up	MyD88/Smad3	[77]
lncRNA-XIST	Mice A549	Up	miR-101-3p	[78]
lncRNA-ATB	Beas-2B, A549	Up	miR-200c	[79]
LOC103691771	Rat Rat lung fibroblasts	Up	TGF-β1/Smad2/3	[81]
lnc-SNHG1	Mice MRC-5	Up	miR-326	[82]
MRAK050699	Rat	Up	TGF-β/Smad3	[83]
lncRNA MALAT1	Mice	Up	miR-503/miR-145	[56, 84]
lncRNA UCA1	Mice A549	Up	miR-204-5p	[85]
lncRNA ADGRG3	Patients	Down		[87]

### circRNA

circRNA is a circular RNA molecule produced by a covalent combination of the 3′ end and 5′ end driven by reverse shear or lasso. Due to its end-to-end circular structure, circRNA is difficult to be degraded by ribonucleases and is more stable than linear RNA. Generally, circRNAs can be divided into exonic circRNAs, intronic circRNAs, and exon–intron circRNAs. There are 4 mechanisms of circRNA: (1) sponge adsorption of miRNAs; (2) regulation of RNA-binding protein expression by adsorption of protein factors; (3) direct regulation of their parent gene expression by cis or by trading; and (4) encode proteins [88].

#### In vitro and in vivo experiments

Cheng et al. uncovered that circ-012091-regulated PPP1R13B played a key role in the development of silicosis by promoting the proliferation and migration of lung fibroblasts through endoplasmic reticulum stress and autophagy [89]. Similarly, Cao et al. found that endoplasmic reticulum stress induced by silica exposure promoted the development of silicosis, which was related to the increased expression of sigma-1 regulated by circHIPK2 [90]. In addition, circHIPK3 could enhance the expression of forkhead box K2 (FOXK2) via sponging miR-30a-3p, thereby facilitating fibroblast glycolysis and activation, while miR-30a-3p overexpression or FOXK2 knockdown blocked fibroblast activation and abrogated the profibrotic effects of circHIPK3 [91]. Yang et al. found that circZC3H4 could act as ceRNA in RAW264.7 cells through sponge adsorption to regulate miR-212 activity, thereby inhibiting the silencing effect of miR-212 on the target gene zinc finger CCCH-type containing 4 protein (ZC3H4) and increasing the expression level of ZC3H4 protein, which in turn promoted silica exposure-induced activation of alveolar macrophages, and the activated macrophages promoted the proliferation and migration of lung fibroblasts [92]. Another study found that after exposure to silica particles, ZC3H12A-mediated ubiquitination in RAW264.7 cells decreased the expression of the downstream host gene HECTD1, which participated in the polar transformation of lung macrophages, released inflammatory cytokines, and accelerated the process of fibrosis [93]. Furthermore, Chu et al. found that silica-induced autophagy was reversed by overexpression of circHECTD1 or HECTD1 knockdown in HPF-a cells, and silica-induced fibroblast activation, proliferation, and migration were restored via downstream autophagy. These data provided new insight into the potential of circHECTD1/HECTD1 as therapeutic targets for silicosis [94].

circRNA CDR1as stimulated by silica could sponge miR-7 to release TGFBR2 and play a significant role in the process of pulmonary fibrosis by promoting EMT process [95]. cirCECTD1 was found by Fang et al. to promote EMT in mouse lung microvascular endothelial cells when exposed to silica particles. The possible regulatory mechanism was that increased expression of cirCECTD1 in response to silica exposure reduced the expression of the host gene HECTD1 by competitively binding to the precursor mRNA, thus promoting EMT, triggering proliferation and migration of endothelial cells, and finally leading to irreversible lung fibrosis [96].

#### Human experiment

Cheng et al. observed that has_circ_0058493 was highly expressed in silicosis patients through RNA-sequencing. In particular, this study also found that hsa_circ_0058493 knockdown inhibited the expression of fibrotic molecules by influencing the EMT process [97] (Table 3).

**Table 3:** The main circRNA changes involved in silicosis

circRNA	Tissue/Cell/Patients	Expression level	Target gene	References
circ-012091	L929, HPF-a	Up	PPP1R13B	[89]
circHIPK2	HPF-a	Up	sigma-1	[90]
circHIPK3	MRC-5, NIH/3T3	Up	miR-30a-3p/FOXK2	[91]
circZC3H4	RAW264.7	Up	miR-212	[92]
circHECTD1	HPF-a	Down	HECTD1	[94]
circRNA CDR1as	Mice HBE, A549, MRC-5, NIH-3T3	Up	miR-7	[95]
cirCECTD1	Mice Pulmonary tissues of silicosis patients MML1, RAW264.7	Up/Down	HECTD1	[96]
hsa_circ_0058493	Patients	Up		[97]

Among the ncRNA studies related to silicosis, miRNA is the most widely studied. A great number of miRNA microarrays and RNA-sequencing in human and animal peripheral blood and lung tissues provide biomarkers for the screening and diagnosis of silicosis. Simultaneously, a lot of studies have explored the significant role of miRNAs, most of which focus on the regulation of miRNAs on EMT and fibrosis-related target genes. And the antifibrosis effect of miRNAs in silicosis is mainly discussed in cell and animal experiments. Besides, due to the late start of the research on lncRNAs and circRNAs, the pathogenic mechanism of them in silicosis has not been fully studied, and the application in the treatment of silicosis remains in the exploratory stage. In particular, looking for ncRNAs that can be used as targets will provide novel directions for the treatment of silicosis. Meanwhile, studying integrated RNA interaction networks may further reveal the mechanisms of the occurrence and development of silicosis.

## Histone modifications

Histones (H1, H2A, H2B, H3, and H4) are essential proteins that closely related to DNA and promote DNA compaction in the nucleus of eukaryotic cells. Histone modification is a covalent modification process, in which the N-terminal amino acid residues of histones are methylated, acetylated, phosphorylated, ubiquitinated and adenosine diphosphate (ADP)-ribosylated under the catalysis of relevant enzymes. The post-translational modifications affect chromatin compaction and thus access to DNA by regulatory proteins, affecting DNA recombination, replication, repair, and regulation of gene expression [13]. Zhang et al. observed the level of extracellular histones in the plasma of silicosis cases and found that the plasma level of H4 was significantly correlated with the stage of silicosis, indicating that extracellular histones played a vital role in the progression of fibrosis in silicosis [98].

Histone methylation can increase the affinity of histones for DNA, change the interaction between histone tails and DNA or chromatin-associated proteins, and thus transform the structure and function of ribosomes [99]. Histone methylations are catalyzed by histone methyltransferases (HMTs), which are able to add methyl groups provided by S-adenosylmethionine to their target residues. Generally, methylation can be added to the ε-amino group of lysine in the form of mono- (me), di- (me2) or tri- (me3), while arginine methylation can be mono-methylated (me), di-methylated symmetrically (me2s) or asymmetrically (me2a) [100]. By establishing an in vitro model of silicosis, Liu et al. found that there were significant changes in the methylation levels of whole-genome proteins H3K4 and H3K27 before and after transdifferentiation of lung fibroblasts exposed to silica, and the main changes were H3K27 demethylation and H3K4 methylation [18].

Histone acetylation facilitates the dissociation of DNA from histone octamers and the specific binding of transcription factors to DNA binding sites, which loosens chromatin and activates transcription. Conversely, insufficient histone acetylation or inadequate histone deacetylation leads to dense chromatin structure, blocking gene transcription sites, and thus inhibiting gene expression. Generally, histone deacetylases (HDACs) play an essential role in the dysregulation of histone acetylation/deacetylation. Zhang et al. found that exposure of human embryonic lung fibroblasts (helf) to quartz increased the acetylation levels of lysine, histone H2B (lys5/12), H3 (Lys9/14), and H4 (lys12) [101]. In contrast, the methylation level of arginine at position 2 of histone H3 decreased, and ceruloplasmin could reverse the quartz exposure-induced histone acetylation and methylation changes [101].

The role of histone modifications in silicosis has rarely been reported. However, the above studies provide clues for further research on the role of histone modifications in the pathogenesis of silicosis and its treatment and prognosis.

## Conclusions

Silicosis is an occupational disease caused by the inhalation of crystalline silica, which is characterized by diffuse fibrosis of lung tissues and the formation of silicon nodules [102]. During the formation of silicosis, disease-specific triggers can cause inflammation, myofibroblast activation, and the activation of a profibrotic positive feedback loop, leading to the continuous development of fibrosis [103]. Although the pathogenesis of silicosis has not been completely clarified, there are hypotheses that it has certain regulatory mechanisms, including epigenetic regulation. Among the studies of epigenetic regulation related to silicosis, miRNA is the most studied, and the mechanism is relatively complete. Besides, achievements have been made in the research of lncRNA and circRNA in cell signal pathway, transcriptional regulation and function as ceRNA binding to mRNA. However, due to the late start, the relevant research is less than miRNA. And the studies of ncRNA regulation are primarily about EMT and fibrosis-related target genes. Compared with ncRNA, the role of methylation modification and histone modifications in silicosis still needs further exploration.

Previous studies have confirmed that epigenetic regulation can play key roles in the diagnosis and treatment of cancer, which give us great hints [104, 105]. Similarly, methylation modification, differential expression of ncRNAs, and histone modifications can serve as signaling molecules for silicosis and play significant roles in diagnosing and initial assessing the severity of silicosis and its treatment. Exploring epigenetic changes of silicosis is of great value for understanding the pathogenesis, disease surveillance, diagnosis, intervention, and treatment of silicosis. Therefore, using epigenetic factors to find new targets can provide new ideas for the treatment of silicosis. However, the pathogenesis of silicosis is a complex network of response regulation consisting of multiple effector cells and active substances, a large number of studies still need to clarify how epigenetic regulation starts and plays a role in silicosis.

## Data Availability

Not applicable.

## Abbreviations

ADP: Adenosine diphosphate
ALKBH5: alkB homologue 5
ASIR: Age-standardized incidence rate
α‑SMA: α‑Smooth muscle actin
ceRNA: Competitive endogenous RNA
BECN1: Beclin 1
Bcl-2: B-cell lymphoma-2
CHRF: Cardiac hypertrophy-related factor
circRNA: Circular RNA
COL-I: Collagen type I
COL-III: Collagen type III
DEmRNAs: Differently expressed mRNAs
DElncRNAs: Differently expressed lncRNAs
DNMTs: DNA methyltransferases
EIF4A3: Eukaryotic translation initiation factor 4A3
EMT: Endothelial mesenchymal transition
Erdr1: Erythroid differentiation regulator 1
FOXK2: Forkhead box K2
FOXM1: Forkhead box protein M1
FOXP3: Forkhead box P3
HDACs: Histone deacetylases
HMGA2: High mobility group protein 2
HMTs: Histone methyltransferases
lncRNA: Long noncoding RNAs
lncRNA-ATB: LncRNA activated by transforming growth factor-β
MAPK: Mitogen-activated protein kinase
Mbd2: Methyl-CpG-binding domain 2
METTL3: Methyltransferase-like 3
miRNA: MicroRNA
MyD88: Myeloid differentiation factor 88
m^6^A: N^6^-methyladenosine
ncRNA: Non-coding RNA
PARK2: Parkinson’s disease protein 2
piRNA: Piwi-interacting RNA
PTBP1: Polypyrimidine tract-binding protein 1
PTEN: Phosphatase and tensin homolog deleted on chromosome ten
SAM: S-adenyl methionine
Smurf2: Smad ubiquitination regulator 2
snRNA: Small nuclear RNAs
SP1: Specificity protein 1
TGF-β1: Transforming growth factor-β1
TGFBR1: Transforming growth factor-β receptor I
TNFSF14: Tumor necrosis factor superfamily 14
ZC3H4: Zinc finger CCCH-type containing 4 protein
ZEB1: E-box-bound zinc finger protein 1
3′UTRs: 3′ untranslated regions
